# Preparation of magnetic activated carbon fibers@Fe_3_O_4_ by electrostatic self-assembly method and adsorption properties for methylene blue

**DOI:** 10.1098/rsos.240497

**Published:** 2024-07-31

**Authors:** Jia-yi Ye, Man-qing Ye, Ling Zhang, Wen Li, Yan-shan Li, Zhi-wei Fu

**Affiliations:** ^1^ College of Biological and Food Engineering, Guangdong University of Petrochemical Technology, Maoming 525000, People’s Republic of China

**Keywords:** coconut palm fibers, activated carbon, magnetic, nano-Fe_3_O_4_, adsorption dynamics, isothermal adsorption models

## Abstract

Nano-Fe_3_O_4_ was loaded onto coconut-based activated carbon fibres (CACF) using an electrostatic self-assembly method. The effects of the mass ratio of CACF to nano-Fe_3_O_4_, loading time, pH and temperature on the loading effect were investigated and ideal loading conditions were determined. To study the adsorption performance of MACF@Fe_3_O_4_ for methylene blue, the effects of the initial concentration, pH and time on the adsorption were investigated and the working conditions of adsorption were established. MACF@Fe_3_O_4_ was systematically characterized. Adsorption kinetics were investigated under ideal conditions. The ideal loading conditions for MACF@Fe_3_O_4_ were as follows: mass ratio of 1:1, 20 min, pH 9.36, 22.5°C. The saturation magnetization of MACF@Fe_3_O_4_ was 48.2263 emu·g^−1^, which could be quickly separated under an external magnetic field. When the dosage was 0.010 g, the adsorption rate reached 97.29% and the maximum adsorption capacity was 12.1616 mg·g^−1^. The adsorption process conformed to pseudo-first-order kinetics during the first 15 min and pseudo-second-order kinetics during 20–120 min. The equations were $\text{ln(}Q_{e}\text{-}{\text{Q}}_{t}\text{)=2.2394-0.0689t}$ and $\frac{t}{Q_{t}}\text{=0.0774 + 0.5295t}$, respectively. The isothermal adsorption model showed that MACF@Fe_3_O_4_ was more in line with the Langmuir model, indicating that the adsorption process was mainly monolayer adsorption. The thermodynamic analysis results showed that the adsorption process of MB by MACF@Fe_3_O_4_ was an endothermic process. In this study, MACF@Fe_3_O_4_ with high adsorption capacity and easy separation from coconut palm fibres has good application prospects in the field of adsorption, which can promote the high-value utilization of coconut palms.

## 1. Introduction

With the continuous increase of coconut production in southern China, the total amount of coconut palm fibre by-products is also increasing. The coconut palms are natural filamentous fibres extracted from coconut shells. They are moisture-proof, antibacterial, harmless and recyclable. At present, the degree of utilization of coconut palm fibres is low, and there is an urgent need to develop high-value materials, such as bio-activated carbon fiber (BACF). Coconut palm fibre is rich in lignin, hemicellulose and cellulose, which is a good raw material for the preparation of activated carbon (AC). AC is considered to be an ideal adsorbent in wastewater treatment systems. Especially the problem of printing and dyeing wastewater treatment, printing and dyeing wastewater contains methylene blue (MB), malachite green, indigo carmine and so on [1–4]. However, it is difficult to separate and recover AC after use [5], especially powdered AC, which must be separated from water by filtration, coagulation, flocculation, clarification and sedimentation [6], resulting in increased costs. This industrial problem is urgently solved. Recently, by loading ferrum (Fe), Cobalt (Co), Nickel (Ni) and other magnetic metals or their oxides to modify AC [7–12], the separation of adsorbent can be achieved under the action of an external magnetic field. However, applications of Co and Ni in medicine are limited. In contrast, ferrosoferric oxide (Fe_3_O_4_) is an iron compound that can be magnetized and is the most commonly used non-toxic magnetic material. The reported carbon (C)-based adsorbents loaded with Fe_3_O_4_ include magnetic graphene [13], magnetic biochar [14], magnetic covalent organic frameworks [15], magnetic metal organic frameworks [16], etc. However, there are few reports on the study of coconut-based activated carbon fibres (CACF) [17] loaded with nano-Fe_3_O_4_. The preparation of ferromagnetic AC composites with coconut palm fibre as biomass raw material is an important way to promote the comprehensive utilization of coconut processing by-products and increase the added value of products.

Therefore, in this article, magnetic activated carbon fibre @ Fe_3_O_4_ (MACF@Fe_3_O_4_) was prepared by electrostatic self-assembly method using CACF as raw material and systematically characterized. The ideal preparation conditions of MACF@Fe_3_O_4_ and the adsorption conditions of MB were determined. The adsorption kinetics, adsorption isotherms and adsorption thermodynamics were studied.

## 2. Methods

### 2.1. Material and reagents

Coconut palm fibres were provided by Shanghai Zhao-Kuo Purification Equipment Co., Ltd., and nano-Fe_3_O_4_ was provided by Shanghai Aladdin Biochemical Technology Co., Ltd. The remaining conventional reagents were analytical reagents.

### 2.2. Preparation of MACF@Fe_3_O_4_

#### 2.2.1. Preparation of CACF

Based on the authors' previous research [17], a sufficient amount of coconut palm fibres was placed in a crucible and sent to a high-temperature box-type resistance furnace for carbonization at 600°C for 120 min. After cooling to room temperature, samples were removed and ground into powder. Carbonized coconut palm fibres (10.0 g) were weighed and mixed with KOH at a ratio of 1:2 and 75% ethanol was added. The mixture was heated and stirred until the liquid was completely volatilized. The carbonized coconut palm fibres that fully absorbed KOH were placed in a crucible, in a high-temperature vacuum tube furnace and activated at 900°C for 150 min under nitrogen. The crude product was rinsed several times with distilled water, and CACF was obtained after drying.

#### 2.2.2. Single factor experiment

Referring to the electrostatic self-assembly method of Wei *et al.* [18] 0.010 g of CACF was weighed according to different mass ratios, and nano-Fe_3_O_4_ was added to 50 ml of distilled water. The pH of the mixture was adjusted to a fixed value and the mixture was allowed to stand at a certain temperature for a certain period. The effects of mass ratio, preparation time, pH and preparation temperature on the adsorption effect were investigated. After filtration, the residue was dried at 70°C for 120 min to obtain MACF@Fe_3_O_4_.

The 0.010 g MACF@Fe_3_O_4_ was put to 50 ml of 4.0 mg·l^−1^ MB and shaken at 25°C for 60 min under natural pH (natural pH refers to the state when pH was not adjusted). The change in the dye concentration was monitored using a *UVmini-1280* ultraviolet–visible spectrophotometer (Shimadzu Corporation).

#### 2.2.3. Evaluation of magnetic separation effect

The 0.020 g MACF@Fe_3_O_4_ was added to a sample bottle, 20 ml of distilled water was added and the mixture was shaken to simulate the adsorption. A circular magnet with an outer diameter of 80 mm was placed beside the sample bottle, and the magnetic separation of MACF@Fe_3_O_4_ was observed after approximately 1 min.

#### 2.2.4. Measuring method

A series of aqueous solutions of MB at different concentrations were prepared, and the concentration–absorbance curve at 664 nm was determined. The standard curve equation was *A* = 0.2305C − 0.0030 (*R*^2^ = 0.9989). The adsorption capacity (*Q*_e_) was calculated using equation (2.1) and the adsorption rate (*D*) was calculated using equation (2.2):


(2.1) $$Q_{e}=\left(C_{0}-C_{e}\right)\times \frac{V}{m}$$

(2.2) $$D=\frac{C_{0}-C_{e}}{C_{0}}\times 100\%$$

where *C*_e_ (mg·l^−1^) is the residual concentration of MB, *C*_0_ (mg·l^−1^) is the initial concentration of MB, *V* (l) is the volume of the MB solution and *m* (g) is the mass of the MACF@Fe_3_O_4_.

### 2.3. Structural characterization

The external surfaces of the CACF and MACF@Fe_3_O_4_ were observed and analysed using a *Regulus8220* SEM (Hitachi Limited), and the elemental composition was analysed using EDS. A *Vertex 70* FTIR was used to analyse the chemical groups of CACF, nano-Fe_3_O_4_ and MACF@Fe_3_O_4_ in the wave number of 400–4000 cm^−1^. Magnetic hysteresis loop analysis of nano-Fe_3_O_4_ and MACF@Fe_3_O_4_ prepared with different mass ratios was performed using a Physical Property Measurement System (PPMS). The crystal forms of CACF and MACF@Fe_3_O_4_ were analysed between 2θ = 20°−80° using the *Ultima IV* X-Ray Diffractometer of Rigaku Corporation, Japan. The pyrolysis mass loss of CACF and MACF@Fe_3_O_4_ was analysed by *STA449F3* synchronous thermal analyser of NETZSCH Company in Germany.

### 2.4. Study on adsorption properties of MACF@Fe_3_O_4_

#### 2.4.1. Optimization of adsorption conditions

The 50 ml of MB with different initial concentrations was taken, 0.010 g MACF@Fe_3_O_4_ was added, the pH of the mixture was adjusted to a fixed value and the mixture was oscillated at 25°C for 60 min. The supernatant was collected and the concentration of the MB solution was monitored using a *UVmini-1280* ultraviolet–visible spectrophotometer. The effects of the initial MB concentration, pH and adsorption time on adsorption performance were investigated.

#### 2.4.2. Study on recycling performance

Under the optimized ideal working conditions, the MACF @ Fe_3_O_4_ after one use was separated by suction filtration, dried in vacuum at 70°C and then reused for 5–6 times. The reaction time was 60 min and the adsorption rate of each use was determined.

### 2.5. Study on adsorption kinetics

Thirty-six samples of 0.010 g of MACF@Fe_3_O_4_ were respectively added to 50 ml of 5.0 mg·l^−1^ MB and oscillated at a constant temperature of 25°C (150 r·min^−1^). The amount of MACF@Fe_3_O_4_ adsorbed was measured with the change in adsorption time, and relevant kinetic data were obtained. The adsorption kinetics [19] of MB on MACF@Fe_3_O_4_ were fitted by the pseudo-first-order, pseudo-second-order and intraparticle diffusion equations which were calculated using equations (2.3)–(2.5):


(2.3) $$In(Q_{e}-Q_{t})=In-K_{1}t,$$

where *Q*_*t*_ (mg·g^−1^) is the adsorption capacity of MB at time *t* (min), *Q*_e_ (mg·g^−1^) is the amount of adsorption at equilibrium and *K*_1_ is a pseudo-first-order equation constant.


(2.4) $$\frac{\text{t}}{Q_{t}}\text{=}\frac{\text{1}}{K_{\text{2}}Q_{e}^{\text{2}}}\text{+}\frac{t}{Q_{e}},$$

where *Q*_*t*_ (mg·g^−1^) is the adsorption capacity of MB at time *t* (min), *Q*_*e*_ (mg·g^−1^) is the amount of adsorption at equilibrium and *K*_2_ is a pseudo-second-order equation constant.


(2.5) $$Q_{t}\text{=}K_{\text{id}}^{\frac{\text{1}}{\text{2}}}+C,$$

where *K*_*id*_ is a intraparticle diffusion equation constant, C (mg·g^−1^) is an empirical constant and *Q*_t_ (mg·g^−1^) is the MB adsorption capacity at time *t* (min).

### 2.6. Study on adsorption isotherm

The adsorption isotherm describes the relationship between the adsorption capacity and the equilibrium concentration at a fixed temperature. Eighteen samples of 0.010 g MACF@Fe_3_O_4_ were added into 50 ml MB solution (5.0, 15.0, 20.0, 30.0, 40.0 and 50.0 mg·l^−1^), respectively. The samples were shaken at 25, 35 and 45°C (150 r·min^−1^), and the absorbance value was measured after 150 min. The adsorption isotherms of MB on MACF@Fe_3_O_4_ were calculated by the Langmuir model and Freundlich model. The corresponding equations are as follows:


(2.6) $$\frac{C_{e}}{Q_{e}}=\frac{1}{Q_{m}K_{\text{L}}}+\frac{C_{\text{e}}}{Q_{m}},$$

(2.7) $$\ln Q_{e}\text{=ln}\;K_{\text{F}}\text{+}\frac{\text{1}}{\text{n}}\ln C_{e},$$

where *C*_e_ (mg·l^−1^) is the concentration of MB after 150 min, *Q*_e_ (mg·g^−1^) is the amount of adsorption at equilibrium, *Q*_m_ (mg·g^−1^) is the maximum adsorption capacity, *K*_L_ is constant of the Langmuir model, *K*_F_ is constant of the Freundlich model and $\frac{1}{n}$ is the constant of adsorption strength.

### 2.7. Adsorption thermodynamics

Thermodynamic parameters can reflect the energy changes in the adsorption process, mainly including adsorption entropy variable (Δ*S*°), adsorption enthalpy variable (Δ*H*°) and Gibbs free energy variable (Δ*G*°), which are helpful to judge whether the adsorption reaction can occur on its own and its adsorption nature. According to the adsorption isotherm equilibrium data at different temperatures, the thermodynamic analysis was carried out by using Van 't Hoff equation and thermodynamic relationship:


(2.8) $$\Delta G=-RTlnK_{c,}$$

(2.9) ΔG=ΔH−TΔS,

(2.10) $$K_{c}\text{=}\frac{Q_{e}}{C},$$

(2.11) $$\ln K_{c}\text{=}\frac{\Delta S}{R}-\frac{\Delta H}{RT},$$

where *K*_c_ is the partition coefficient, *Q*_e_ (mg·g^−1^) is the amount of adsorption at equilibrium, *C* (mg·l^−1^) is the concentration of remaining solution, *R* (8.314 J·mol^−1^·K^−1^) is the gas molar constant and *T* (K) is Fahrenheit degree.

## 3. Results

### 3.1. Preparation results

#### 3.1.1. Effect of mass ratio on adsorption and magnetic separation

**Figure 1.**
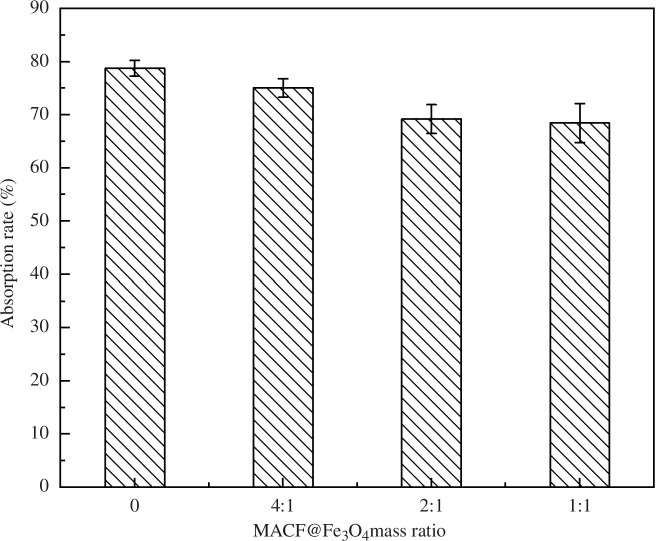
Effect of mass ratio on adsorption rate.

**Figure 2.**
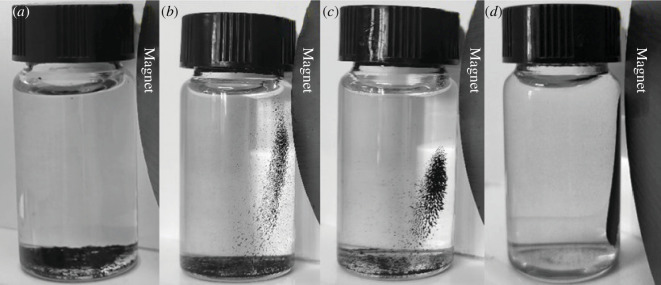
Effect of mass ratio on magnetic separation. (*a*) CACF. (*b*) CACF:Fe_3_O_4_ = 4:1. (*c*) CACF:Fe_3_O_4_ = 2:1. (*d*) CACF:Fe_3_O_4_ = 1:1.

The effect of MACF@Fe_3_O_4_ which was prepared using mass ratios of CACF to nano-Fe_3_O_4_ of 4:1, 2:1 and 1:1, on the adsorption rate and adsorption capacity of MB is shown in figure 1, and the effect on magnetic separation is shown in figure 2. Figure 1 shows that the MB adsorption rate of MACF@Fe_3_O_4_ decreased with increasing nano-Fe_3_O_4_ addition. When nano-Fe_3_O_4_ was not loaded, the adsorption rate of MB by the CACF was 78.74%. At a mass ratio of 1:1, the adsorption rate of MACF@Fe_3_O_4_ was 68.44%, which was 13.08% lower than that of the CACF. Simultaneously, the decrease in the rate of MB absorption by MACF@Fe_3_O_4_ began to slow, indicating that the adsorption of MB by MACF@Fe_3_O_4_ tended to be balanced, and the addition of excess nano-Fe_3_O_4_ increased the preparation cost. Figure 2 shows that with an increase in nano-Fe_3_O_4_ addition, the magnetic separation of MACF@Fe_3_O_4_ was better. When the mass ratio was 1:1, most of the MACF@Fe_3_O_4_ was attracted to one side by the magnet, and the magnetic separation was better than that of the sample with a mass ratio of 2:1. Wei [18] also adopted the method of electrostatic self-assembly to prepare Fe_3_O_4_@GO particles at a mass ratio of 1:1, which can be easily dispersed in solution and separated using ordinary magnets.

**Figure 3.**
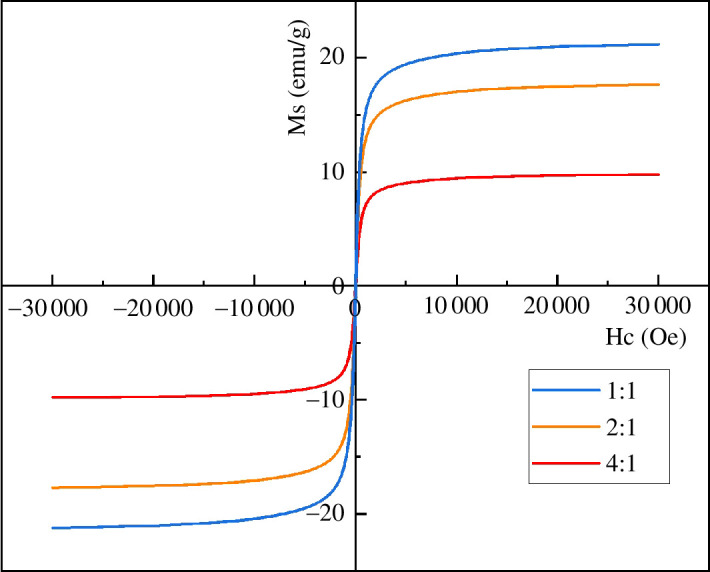
Magnetic hysteresis loops of MACF@Fe_3_O_4_ with different mass ratios.

Figure 3 shows the magnetic hysteresis loop of MACF@Fe_3_O_4_ prepared at different mass ratios. The hysteresis loops of MACF@Fe_3_O_4_ with different mass ratios are *S*-shaped, indicating superparamagnetic characteristics. However, with the decrease in nano-Fe_3_O_4_ addition, the saturation magnetization (Ms) decreased and the magnetism gradually weakened. In summary, to enhance the magnetic separation ability of CACF and maximize the adsorption effect of MACF@Fe_3_O_4_, as well as possible, the mass ratio of CACF to nano-Fe_3_O_4_ was 1:1.

#### 3.1.2. Effect of preparation time on adsorption and magnetic separation

**Figure 4.**
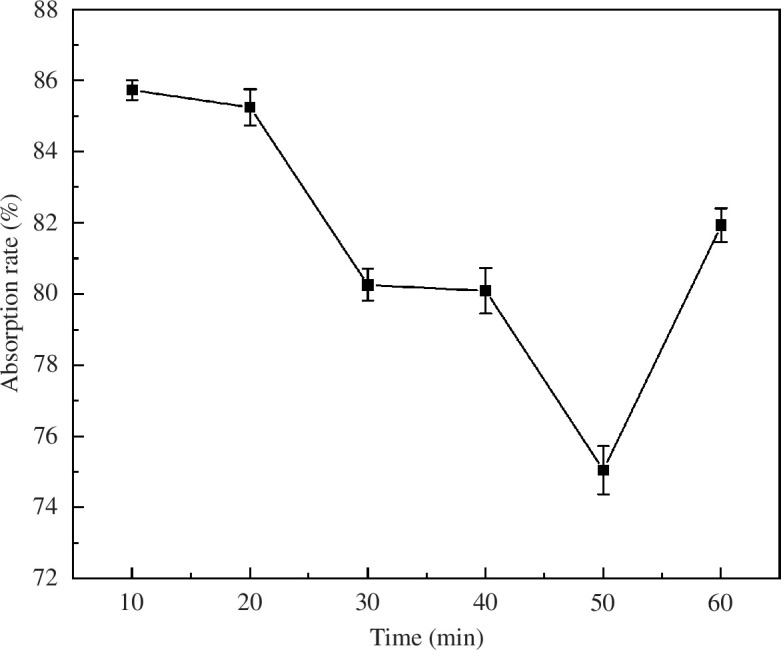
Effect of preparation time on the adsorption rate.

Figure 4 shows the effect of the preparation time of MACF@Fe_3_O_4_ on the adsorption rate of MB.

It can be seen that the MB adsorption by MACF@Fe_3_O_4_ decreased first and then increased. During 10–50 min, the adsorption rate of MB on MACF@Fe_3_O_4_ gradually decreased with increasing preparation time. The sites on the surface of the CACF were gradually occupied by nano-Fe_3_O_4_, so that the adsorption rate decreased when adsorbing MB. However, MACF@Fe_3_O_4_ still had a good adsorption effect, which was consistent with the results of Zhu-qing *et al.* [20]. The MB adsorption rate of MACF@Fe_3_O_4_ increased to 81.94% at 60 min. This may be because some of the nano-Fe_3_O_4_ on MACF@Fe_3_O_4_ was desorbed. Therefore, more sites on MACF@Fe_3_O_4_ could adsorb MB and the adsorption rate increased.

**Figure 5.**
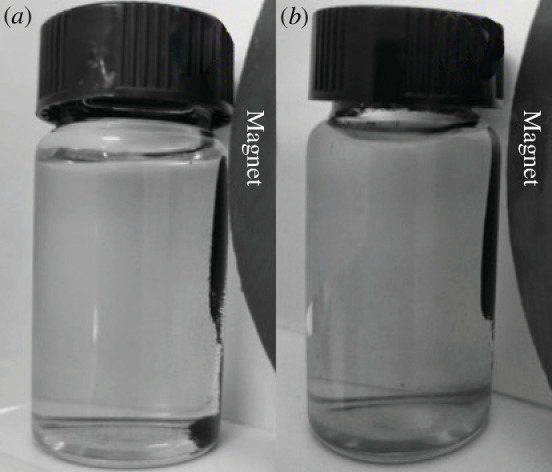
Effect of preparation time on the magnetic separation. (*a*) 10 min. (*b*) 20 min.

Figure 5*a,b* shows the magnetic separation diagrams for the preparation times of 10 and 20 min, respectively. Comparing figure 5*a,b* , it can be seen that MACF@Fe_3_O_4_ with a preparation time of 20 min could be cleanly separated by the magnet, while MACF@Fe_3_O_4_ with a preparation time of 10 min had a poor magnetic separation effect. As shown in figure 5*b*, many MACF@Fe_3_O_4_ powders remained floating in the sample bottle and were not attracted to the magnet. In summary, a preparation time of 20 min had a better magnetic separation effect and better retained the adsorption capacity.

#### 3.1.3. Effect of pH on adsorption

MB is an alkaline dye. When ionized in an aqueous solution, the coloured ions are cations (MB^+^). The surface of CACF may contain acidic oxygen (O)-containing groups such as carboxyl groups (—COOH), lactone groups (—COO—) and phenolic hydroxyl groups (—OH) [21]. These acidic groups can be deprotonated under alkaline conditions and dissociated to form carboxyl anions (—COO^—^) and phenolic oxygen anions (—O^—^), which are beneficial for the electrostatic adsorption of MB^+^ by MACF@Fe_3_O_4_. However, under acidic conditions, the dissociation of these acidic groups is inhibited. Han-bing *et al.* [21] found that the microporous structure of coconut shell AC modified with weak acids was better than that of coconut shell AC modified by strong acids. Therefore, the etching of weak acids on the carbon skeleton was beneficial for MACF@Fe_3_O_4_ to improve the adsorption performance of MB. However, an acid or alkali concentration that was too high degraded the mechanical properties of MACF@Fe_3_O_4_. Part of the carbon skeleton of MACF@Fe_3_O_4_ was oxidized, coupled with the etching effect on the carbon skeleton, resulting in a poor pore structure [22] and the adsorption rate was significantly reduced.

**Figure 6.**
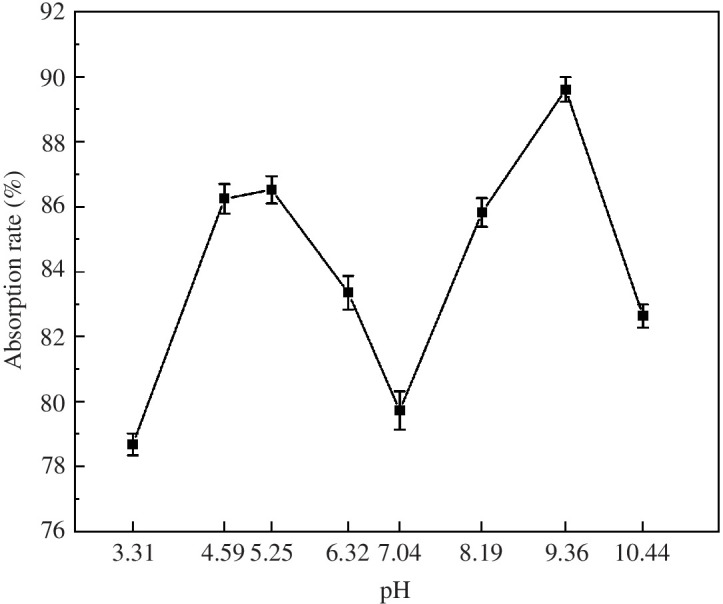
Effect of preparation pH on the adsorption rate.

As shown in figure 6, the preparation pH had a significant effect on the MB adsorption rate of MACF@Fe_3_O_4_. The adsorption rate was 79.73% at the natural pH of 7.04. The adsorption rate of MACF@Fe_3_O_4_ for MB could be improved by adjusting the preparation pH to weakly acidic or alkaline environments, and the adsorption of MACF@Fe_3_O_4_ for MB reached a maximum at pH 9.36. As the acidic or alkaline strength increased, the adsorption rate decreased significantly at pH 3.31 and pH 10.44 and the adsorption rate was the lowest at pH 3.31. Therefore, the optimal preparation pH for MACF@Fe_3_O_4_ was 9.36.

#### 3.1.4. Effect of temperature on adsorption

**Figure 7.**
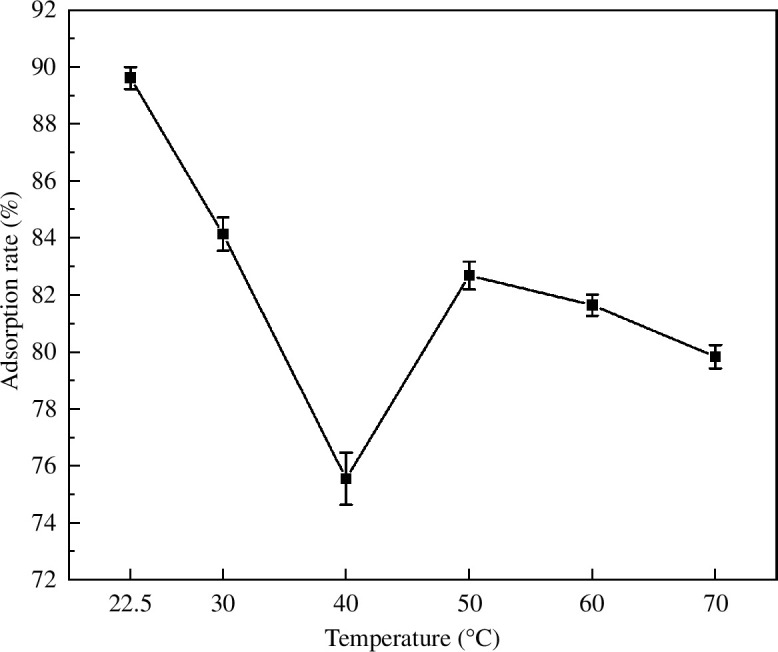
Effect of preparation temperature on the adsorption rate.

Figure 7 shows the effects of different preparation temperatures on the MB adsorption rate. An increase in the temperature inhibited the loading of nano-Fe_3_O_4_ on the CACF. Under the preparation condition of room temperature (22.5°C), the adsorption rate was 89.62%. The lowest adsorption rate was 75.56%. At the preparation temperature of 50°C, the adsorption rate increased to 82.69% and continued to decrease with increasing temperature. Xian-hang *et al.* [23] found that the effect of temperature on the desorption effect was reflected in two aspects, the unfavourable factor is the density effect and the favourable one is the diffusion effect, which ultimately depends on the competition between the two. According to our previous research [17], most of the specific surface area and pore volume of CACF are provided by micropores (pore size <2 nm). Therefore, CACF are advantageous for the adsorption of small-molecule substances. However, the thermal motion of the water molecules in the solution increased and the density of nano-Fe_3_O_4_ in the solution decreased when the preparation temperature increased. The density effect caused by the increase in temperature was greater than the diffusion effect, which was more conducive to the loading of nano-Fe_3_O_4_ by the CACF. After loading, the adsorption sites on the surface of MACF@Fe_3_O_4_ were occupied and the adsorption rate of MB decreased. When the preparation temperature was 50°C, the thermal motion of the water molecules was more intense and the diffusivity increased. At this time, the diffusion effect was greater than the density effect, and nano-Fe_3_O_4_ was desorbed. The surface adsorption sites of the prepared MACF@Fe_3_O_4_ were restored to the no-load state, thereby increasing the adsorption rate of MB. As the preparation temperature continued to increase, it was not conducive to the structural stability of MACF@Fe_3_O_4_, and the MB adsorption rate continued to decline. In summary, the ideal preparation temperature is 22.5°C.

### 3.2. Microstructure analysis

#### 3.2.1. s.e.m. analysis

**Figure 8.**
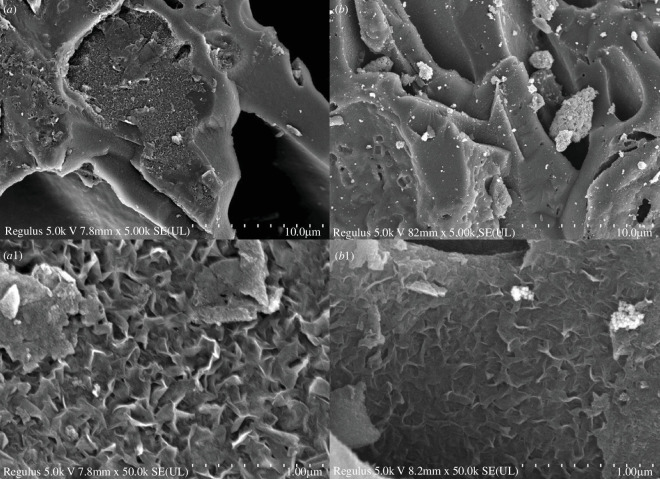
The s.e.m. images of CACF and MACF@Fe_3_O_4_ at different magnifications. (*a****,****a*_1_) CACF. (*b*,*b*_1_) MACF@Fe_3_O_4_. (*a*,*b*) 5000 times. (*a*_1_,*b*_1_) 50 000 times.

From figure 8*a,b*, it can be seen that the surface of the CACF activated by KOH was clean and the pores were clear. Overall, the holes in the CACF were arranged and the cross-section presented an irregular honeycomb pore structure. The developed pore structure increased the surface area and enhanced adsorption, which was conducive to the loading of nano-Fe_3_O_4_. Figure 8*a1*,*b1* show MACF@Fe_3_O_4_ prepared at a mass ratio of 1:1 and preparation time of 20 min (pH 9.3, 25°C). The surface of MACF@Fe_3_O_4_ became rough and the sizes of holes were significantly smaller. This structure endowed MACF@Fe_3_O_4_ with magnetic separation characteristics while retaining its adsorption capacity for dye molecules to a large extent. The nano-Fe_3_O_4_ was uniformly distributed in the form of particles, indicating that it was successfully attached to the surface of the CACF.

#### 3.2.2. EDS analysis

**Figure 9.**
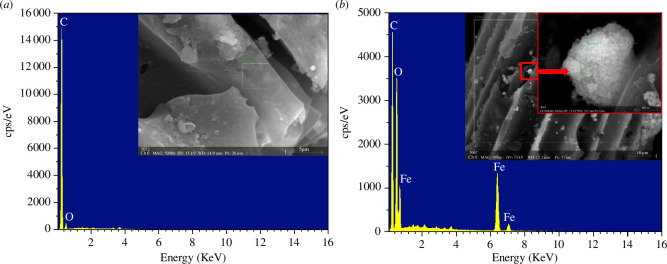
EDS images of CACF and MACF@Fe_3_O_4_. (*a*) CACF. (*b*) MACF@Fe_3_O_4_.

The EDS and surface elemental composition analyses of CACF and MACF@Fe_3_O_4_ are shown in figure 9*a,b* and table 1. As shown in table 1, the elements contained in the CACF were C and O, accounting for 83.72% and 15.23%, respectively. Possible reasons are as follows. First, there were some O-containing functional groups on the surface of the CACF after the KOH treatment. Second, the O in the air was adsorbed by the CACF. No iron was detected on the CACF, whereas MACF@Fe_3_O_4_ contained a large amount of C and O, as well as a certain amount of iron. Iron was loaded onto the CACF in the form of oxide, indicating that nano-Fe_3_O_4_ was successfully loaded onto the CACF.

**Table 1.** Elemental composition of CACF and MACF@Fe_3_O_4_.

element	(a）		(b）	
	wt%	At%	wt%	At%
C	77.88	83.72	24.13	42.09
O	18.87	15.23	31.39	41.10
Fe	—	—	43.61	16.36
others	3.25	1.05	0.96	0.45
total	100.00	100.00	100.00	100.00

#### 3.2.3. FTIR analysis

**Figure 10.**
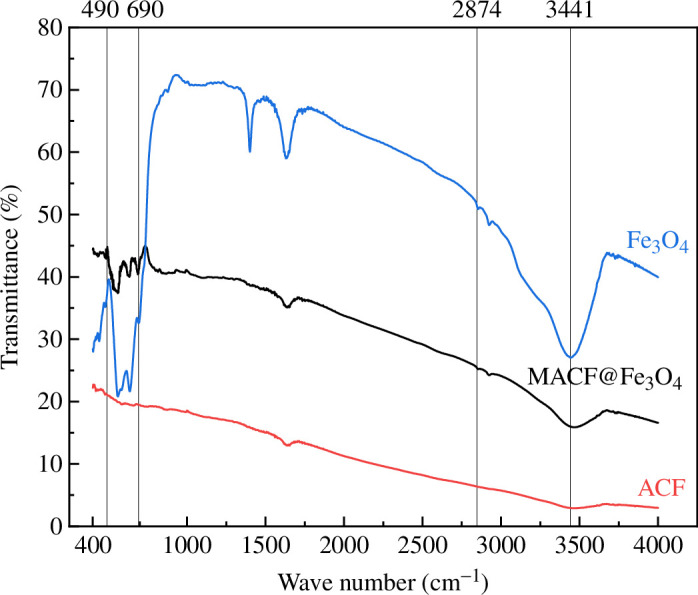
FTIR images of CACF, nano-Fe_3_O_4_ and MACF@Fe_3_O_4_.

Figure 10 shows that compared with CACF, the peak width of MACF@Fe_3_O_4_ at the wave number of 3441 cm^−1^ was significantly enhanced. The broad peak is related to the O—H bond in the hydroxyl or carboxyl groups, indicating that the number of acidic O-containing functional groups increased after modification, which contributed to the adsorption of polar organic molecules. The absorption peaks of MACF@Fe_3_O_4_ between 490 cm^−1^ and 690 cm^−1^ are regarded as the characteristic absorption peak of Fe—O, whereas there was no characteristic peak of Fe—O for the CACF. In the low-frequency region of Fe—O, the absorption peak at 490 cm^−1^ was attributed to the bending vibration of Fe—O—Fe, whereas the absorption peaks between 560 cm^−1^ and 690 cm^−1^ were attributed to the stretching vibration of Fe—O [24,25]. The above characteristic peaks indicate that ferrite compounds were present in the MACF@Fe_3_O_4_ sample.

#### 3.2.4. Magnetic hysteresis loops analysis

**Figure 11.**
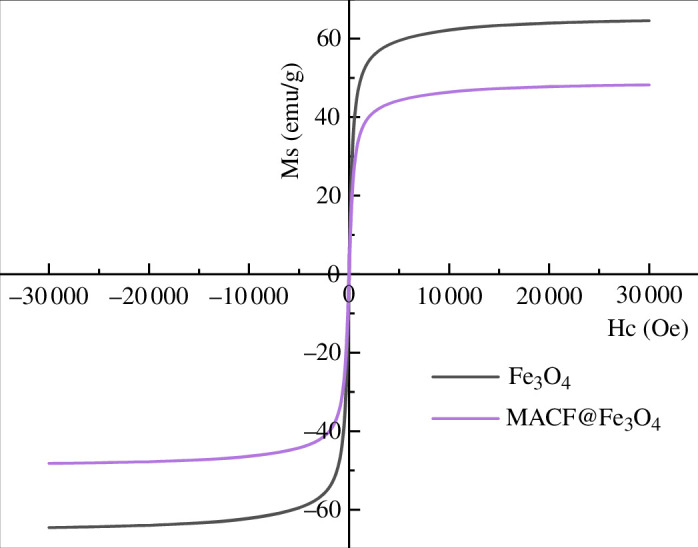
Magnetic hysteresis loops of nano-Fe_3_O_4_ and MACF@Fe_3_O_4_.

The magnetic properties of the samples were studied using a PPMS at room temperature (25°C) and an ±3 *T* external magnetic field. As shown in figure 11, the magnetic hysteresis loops of nano-Fe_3_O_4_ and MACF@Fe_3_O_4_ prepared under ideal conditions were *S*-shaped, and the thermoremanent magnetization and coercive force (Hc) were almost zero, indicating that both were spinel ferrite materials [26]. In addition, the Ms of nano-Fe_3_O_4_ was 64.5678 emu·g^−1^, whereas that of MACF@Fe_3_O_4_ was 48.2263 emu·g^−1^. The Ms value of MACF@Fe_3_O_4_ was smaller than that of nano-Fe_3_O_4_ owing to the presence of non-magnetic CACF in MACF@Fe_3_O_4_. In summary, both nano-Fe_3_O_4_ and MACF@Fe_3_O_4_ have superparamagnetic characteristics, which makes the powdered MACF@Fe_3_O_4_ easy to separate magnetically from the solution. This characteristic contributed to the effective dispersion of MACF@Fe_3_O_4_ to adsorb MB from the aqueous solution and promoted the magnetic separation of MACF@Fe_3_O_4_ from the treated solution using an external magnetic field.

#### 3.2.5. XRD analysis

**Figure 12.**
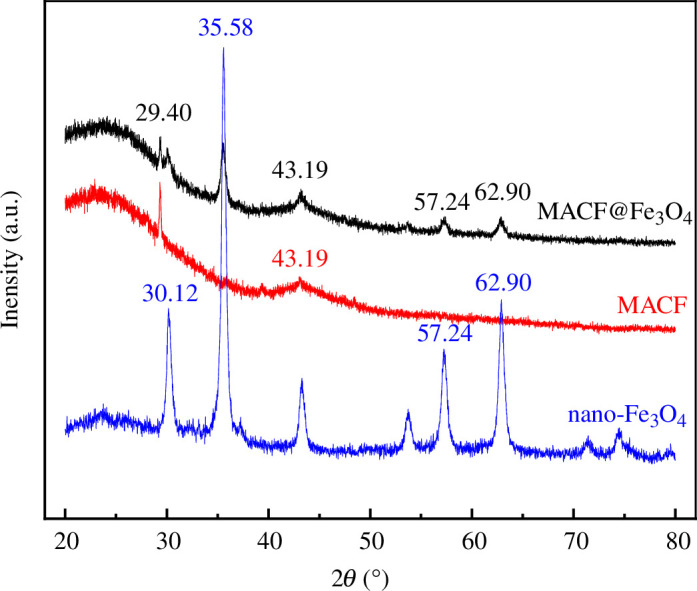
XRD patterns of CACF, nano-Fe_3_O_4_ and MACF@Fe_3_O_4_.

The XRD patterns of CACF before and after loading are shown in figure 12. As shown in figure 12, the pattern of MACF@Fe_3_O_4_ was a broadened diffraction peak, indicating that MACF@Fe_3_O_4_ existed in the form of amorphous C with low crystallinity composed of graphite crystallites. Compared with the CACF before and after loading, at 2θ = 29.40° and 43.19°, there were a graphite crystal (002) and (101) diffraction peaks [27], and both were wide peaks, indicating that the structure of CACF material was mainly in the form of graphite crystallite underdevelopment. Comparing the patterns of MACF@Fe_3_O_4_ and nano-Fe_3_O_4_, it can be seen that the characteristic diffraction peaks of 57.24° (400) and 62.90° (422) appeared in the XRD pattern of MACF@Fe_3_O_4_, indicating that nano-Fe_3_O_4_ was successfully loaded onto the surface of CACF in this experiment [28]. As shown in figure 12, the coverage of MACF@Fe_3_O_4_ was high. The peak at C element (002) became weak, indicating that the main form of iron loaded on CACF was nano-Fe_3_O_4_, and the presence of the load occupied the pores of the coconut shell AC, resulting in the weakening of the C crystal structure [29], thus proving the above point of view.

#### 3.2.6. TG analysis thermogravimetric analysis

**Figure 13.**
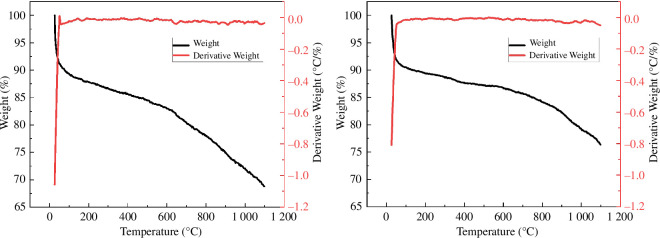
TG/DTG curves of CACF and MACF@Fe_3_O_4_. (*a*) CACF. (*b*) MACF@Fe_3_O_4_.

Figure 13 shows the thermogravimetric (TG) and derivative thermogravimetric (DTG) curves of CACF and MACF@Fe_3_O_4_ from 25°C to 1100°C at a heating rate of 10 *K*·min^−1^, respectively. Throughout the TG and DTG curves, it can be seen that the decomposition process of CACF and MACF@Fe_3_O_4_ was relatively fast, and only one decomposition occurred during the heating process, so there was only one weight loss step. The peak value of the DTG curve appeared at about 50°C, corresponding to the temperature with the largest mass change rate, which was considered to be the water evaporation stage. Subsequently, the remaining mass was large, the decomposition temperature was high and the decomposition process was long. The difference is that the residual mass fraction of MACF@Fe_3_O_4_ was higher after the test, and it was still not balanced at 1100°C, indicating that a large amount of nano-Fe_3_O_4_ was not completely decomposed.

### 3.3. Adsorption results

#### 3.3.1. Effect of initial concentration on adsorption

**Figure 14.**
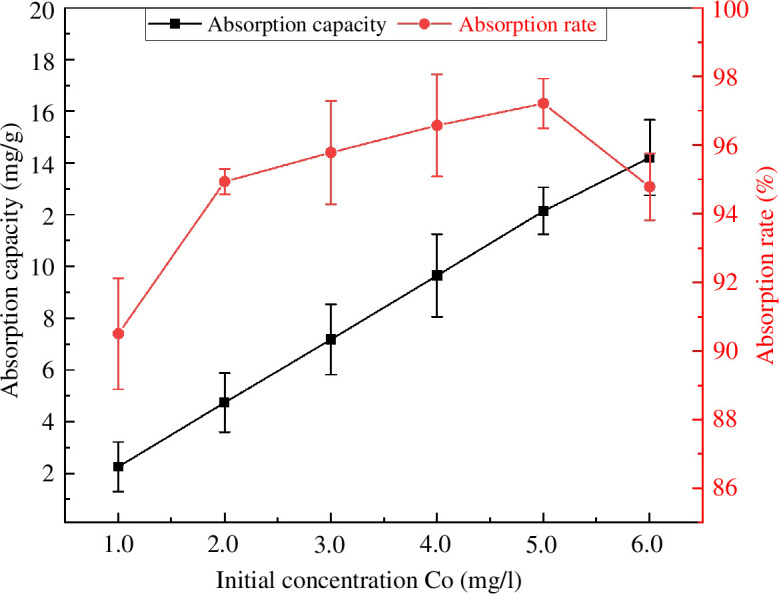
Effect of initial concentration on adsorption capacity and adsorption rate.

Figure 14 shows the effect of the initial dye concentration on the adsorption performance. The adsorption capacity of MACF@Fe_3_O_4_ for MB increased with increasing initial concentration [30], while the adsorption rate first increased and then decreased. At the initial concentration of 5.0 mg·l^−1^, the maximum adsorption rate was 97.22%. When the MB adsorption capacity of MACF@Fe_3_O_4_ at an initial concentration of 6.0 mg·l^−1^ was 14.22 mg·g^−1^, it did not reach equilibrium or showed an equilibrium trend, that is, its adsorption capacity was not saturated. However, it can be seen from the adsorption rate curve that the adsorption rate of MACF@Fe_3_O_4_ for MB began to decline. It can be speculated that if other conditions remained unchanged and the initial concentration continued to increase from 6.0 mg·l^−1^, the adsorption of MB would reach saturation within a certain concentration range.

#### 3.3.2. Effect of pH on adsorption

The molecular structure of MB (p*K*_a_ = 4.52) contains MB^+^ and the pH can affect the number of MB^+^ ions in the solution [31]. When 7.11 < pH < 9.97, the solution was in a weakly alkaline environment, and MB mainly existed in the form of MB^+^. The concentration of MB^+^ increased with increasing pH. At this time, the surface of MACF@Fe_3_O_4_ was negatively charged, resulting in electrostatic adsorption of MB^+^. At 7.11 < pH < 9.97, the adsorption capacity increased significantly with increasing pH. When the pH was 10.85, the adsorption capacity for MB decreased, which was considered to be caused by the destruction of the MACF@Fe_3_O_4_ structure in a strong alkaline environment. In a weakly acidic environment, the number of MB^+^ ions increased with increasing pH, but the surface of MACF@Fe_3_O_4_ was positively charged. Therefore, the effect of electrostatic repulsion was not conducive to MB adsorption by MACF@Fe_3_O_4_. Second, H^+^ and MB^+^ in the solution competed for active sites, therefore, the adsorption capacity decreased with an increase in pH in the range of 5.09 < pH < 7.11. At pH < 4.52, the concentration of MB in the unionized state was higher than that in the ionized state; therefore, MB existed mainly in its molecular form. According to the s.e.m. results, MACF@Fe_3_O_4_ had a rich mesoporous structure. Therefore, when 3.26 < pH < 4.09, a large number of dye molecules entered the pores of MACF@Fe_3_O_4_ mainly through pore filling [32] rather than through electrostatic adsorption.

**Figure 15.**
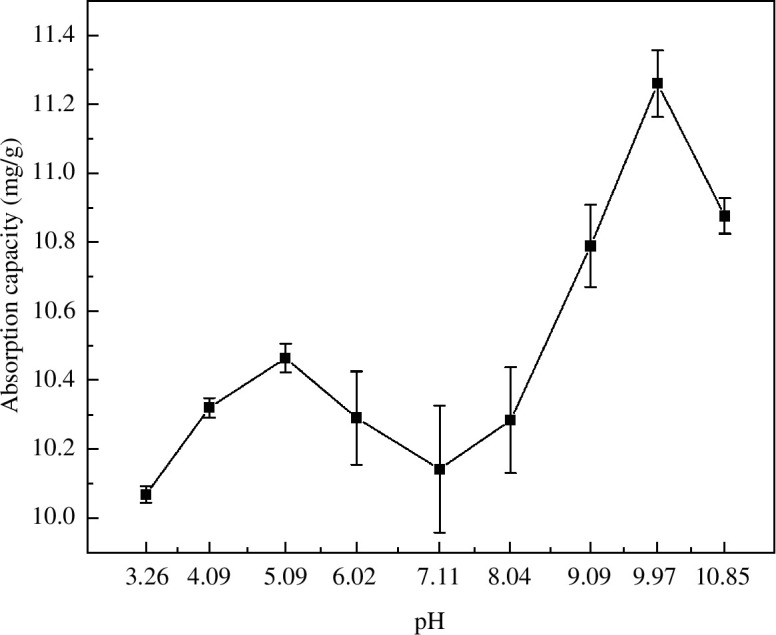
Effect of pH on the adsorption capacity.

As shown in figure 15, adjusting the pH of MB to a weakly acidic or alkaline environment improved the adsorption capacity of MACF@Fe_3_O_4_. The maximum adsorption capacity of MACF@Fe_3_O_4_ for MB was 11.26 mg·g^−1^ at pH 9.97. At a pH of 3.26, the lowest adsorption capacity was 10.07 mg·g^−1^. In summary, the optimum pH for adsorption is 9.97.

#### 3.3.3. Effect of time on adsorption

**Figure 16.**
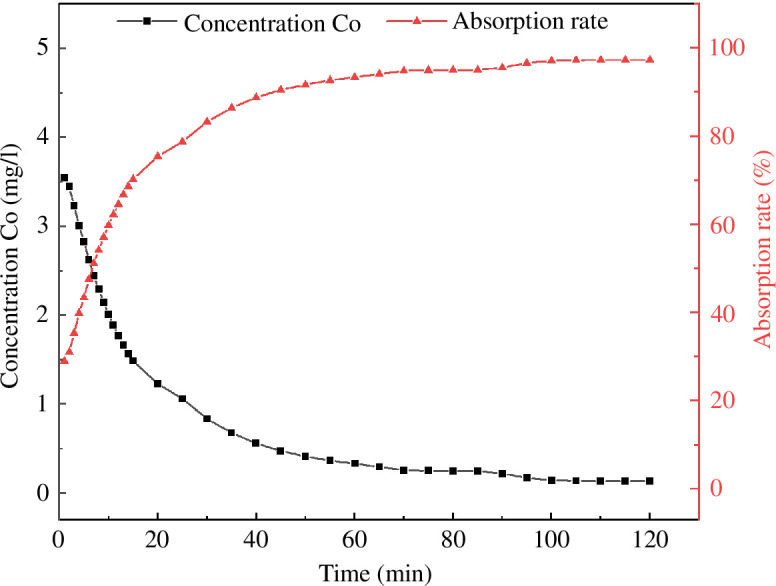
Effect of time on concentration and adsorption rate.

The adsorption capacity of MB with respect to adsorption time is shown in figure 16. The MB adsorption rate of MACF@Fe_3_O_4_ increased with an increase in the adsorption time. The adsorption rate was very fast in the first 10 min, exceeding 50% after 7 min and reaching 90.53% after 45 min. At the beginning of the adsorption time, the concentration of MB was higher, and there were more active adsorption sites and O-containing functional groups on the MACF@Fe_3_O_4_. Therefore, the adsorption capacity gradually increased. In the later stage of adsorption, the growth rate of the adsorption capacity slowed down, and the active adsorption sites and O-containing functional groups on the surface of MACF@Fe_3_O_4_ were gradually occupied. Equilibrium adsorption was reached at 110 min, and the adsorption rate was 97.29%, which showed that MACF@Fe_3_O_4_ had a large adsorption capacity and a fast adsorption rate for MB.

#### 3.3.4. Effect of time on adsorption

**Figure 17.**
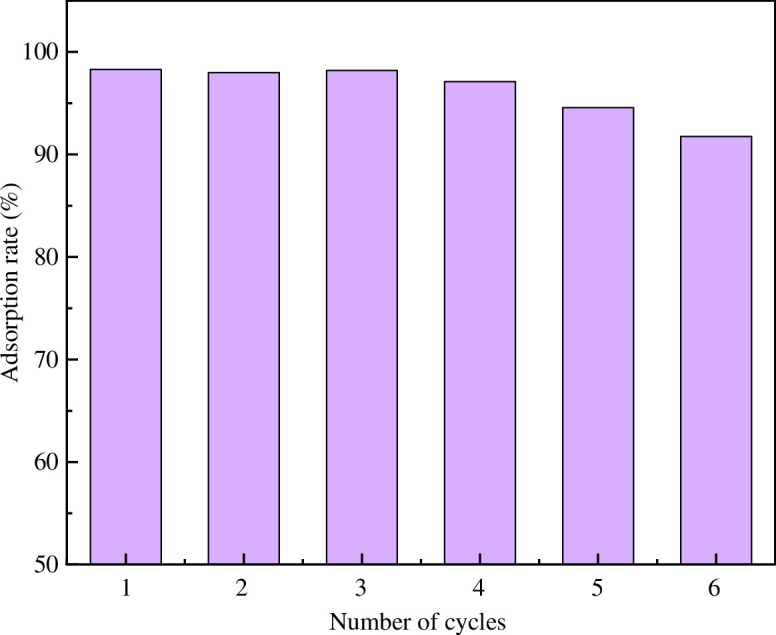
Effect of cycle number on adsorption rate of MB.

Figure 17 shows that MACF@Fe_3_O_4_ shows good adsorption performance in the cycle experiment. After six cycles, the adsorption rate of MB by MACF@Fe_3_O_4_ still remained above 90%, which indicated that MACF@Fe_3_O_4_ had good stability and recycling performance, that is, the recovery rate of magnetic nano-Fe_3_O_4_ was high.

### 3.4. Study on adsorption kinetics

**Figure 18.**
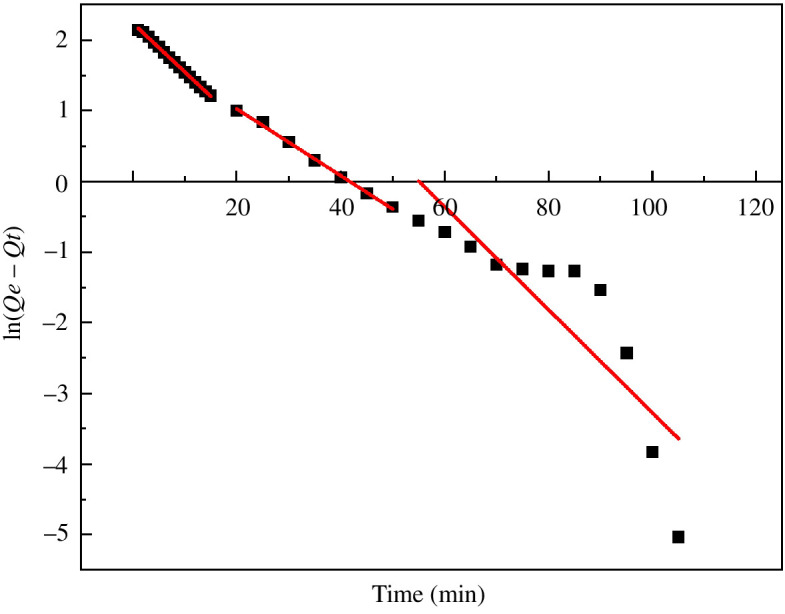
Fitting curve of pseudo-first-order equation.

**Figure 19.**
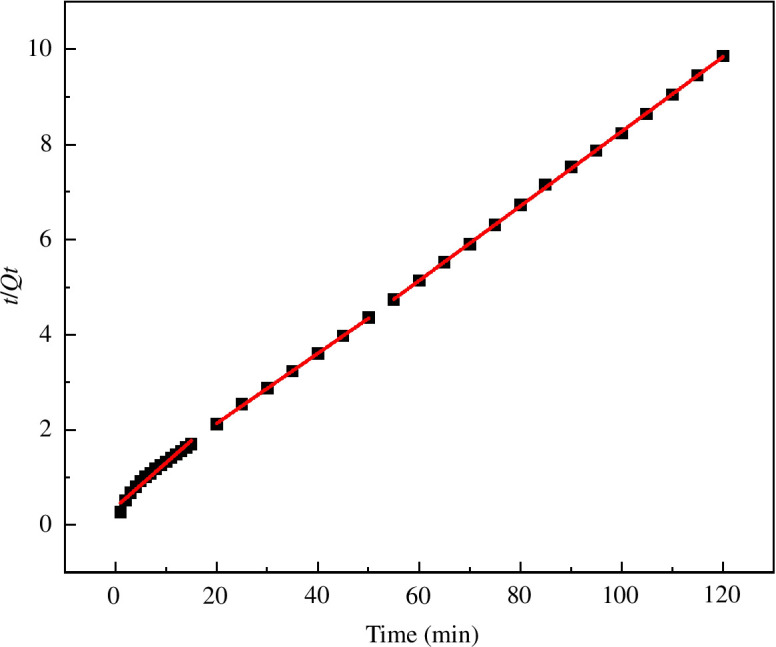
Fitting curve of pseudo-second-order equation.

**Figure 20.**
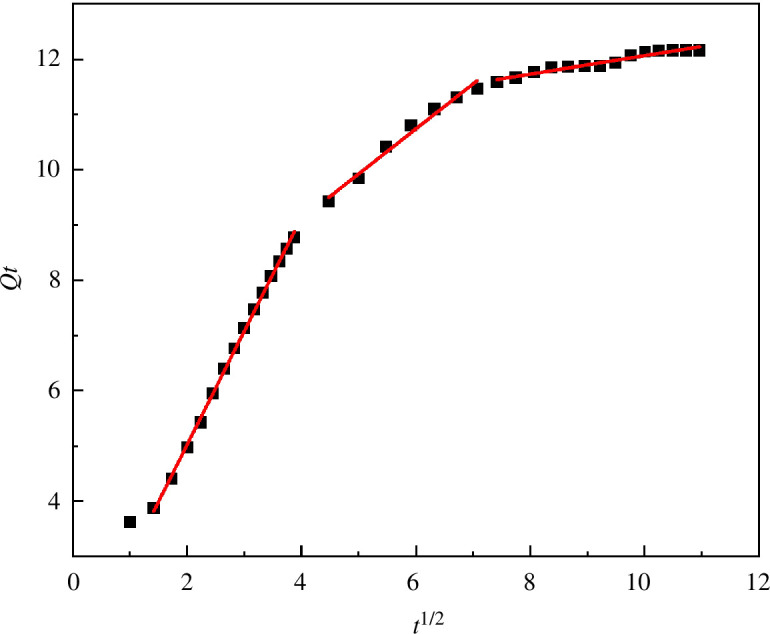
Fitting curve of intraparticle diffusion equation.

The adsorption rate is closely related to the chemical mechanism. By analysing the adsorption kinetics, the adsorption behaviour of the adsorbent and dye can be determined when an adsorption relationship occurs [33]. The adsorption kinetic model is often used to study the change rule of the adsorption rate of the adsorbent to the adsorbate under the influence of different factors. The above results for MACF@Fe_3_O_4_ on the adsorption equilibrium of MB were used for the kinetic adsorption research. The processing time was fitted in three steps: 1–15, 20–50 and 60–120 min. The fitting curve of the pseudo-first-order equation when time (*t*·min^−1^) was plotted against $\text{ln(}{\text{Q}}_{\text{e}}-{\text{Q}}_{t}\text{)}$ is shown in figure 18. The processing time (*t*·min^−1^) was plotted against $\frac{\text{t}}{{\text{Q}}_{\text{t}}}$ , and the pseudo-second-order equation fitting curve is shown in figure 19. The fitting curve for the intraparticle diffusion equation is shown in figure 20, where *t*^1/2^ is plotted against *Q*_*t*_. The relevant parameters for the kinetic model are shown in table 2.

**Table 2.** Kinetic parameters of the adsorption of MB from MACF@Fe_3_O_4_.

kinetic model	*Q*_e, cal_ / (mg·g^−1^)	*K*_1_/（min^−1^）	*K*_2_ / (g·mg^−1^·min^−1^)	*K*_id_ / (g·mg^−1^·min^−0.5^)	*C*/ (mg·g^−1^)	*R* ^2^
pseudo-first-order kinetic						
step 1	9.3875	0.0689				0.9994
step 2	7.2065	0.0473				0.9985
step 3	55.6698	0.0729				0.7382
pseudo-second-order kinetic						
step 1	10.7044		0.2311			0.9686
step 2	13.5722		0.0081			0.9995
step 3	12.7649		0.0139			0.9997
intraparticle diffusion model						
step 1				2.0587	0.9105	0.9726
step 2				0.8146	5.8574	0.9460
step 3				0.1674	10.3951	0.9415

At 1–15 min, the correlation coefficient *R*^2^ of the pseudo-first-order kinetic was 0.9994, and that of the pseudo-second-order kinetic was 0.9686. The fitting degree of the pseudo-first-order kinetic model was higher, indicating that under the same conditions, the pseudo-first-order kinetic model could more accurately describe the dynamic adsorption process of MACF@Fe_3_O_4_ on MB. The reaction rate of MACF@Fe_3_O_4_ was mainly determined by the concentration of MB, which was similar to the pseudo-first-order kinetic reaction. The quasi-first-order kinetic equation was $\text{ln(}{\text{Q}}_{\text{e}}\text{-}{\text{Q}}_{\text{t}}\text{)=2.2394-0.0689t}$. At 20–120 min, the fitting degree of the pseudo-second-order kinetic was higher, indicating that the adsorption process was mainly controlled by chemical action during this period. The pseudo-second-order kinetic equation was $\frac{\text{t}}{{\text{Q}}_{\text{t}}}\text{=0.0774+0.5295t}$. As shown in figure 20 and table 2 that the *K*_id_ of Step 1 was 2.0587, which was greater than that of the other two steps, the correlation coefficient *R*^2^ was the largest, and the fitting degree was the best, indicating that MB diffused more easily inside MACF@Fe_3_O_4_ in the first 15 min. Over time, the active sites of MACF@Fe_3_O_4_ were gradually occupied, and the adsorption process tended to be balanced. The *K*_id_ of Step 3 was the smallest, indicating that this step was the control step of the entire adsorption process. In addition, the C values of the three steps in the intraparticle diffusion kinetic model were all greater than zero, indicating that the intraparticle diffusion kinetic equation did not pass through the origin and that the adsorption process rate was controlled by both intraparticle diffusion and boundary layer diffusion [34].

### 3.5. Study on adsorption isotherm

**Figure 21.**
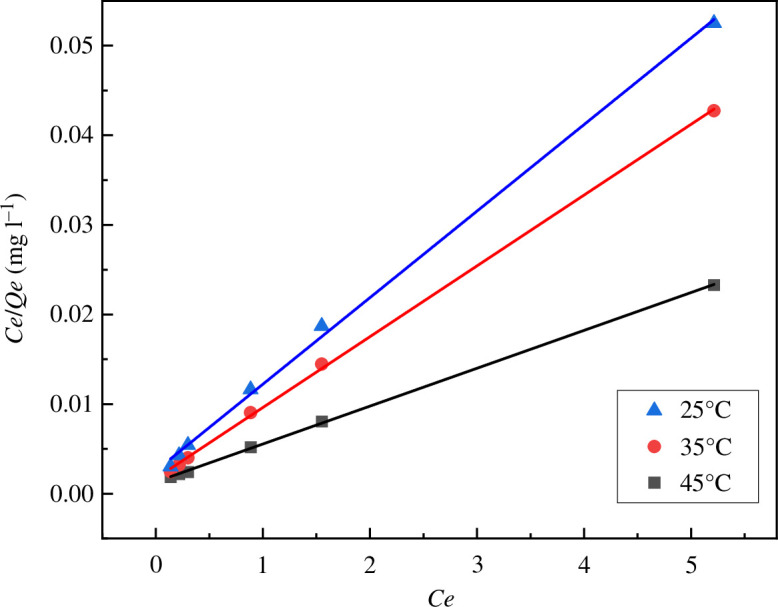
Langmuir isotherm of MACF@Fe_3_O_4_ adsorbing MB.

**Figure 22.**
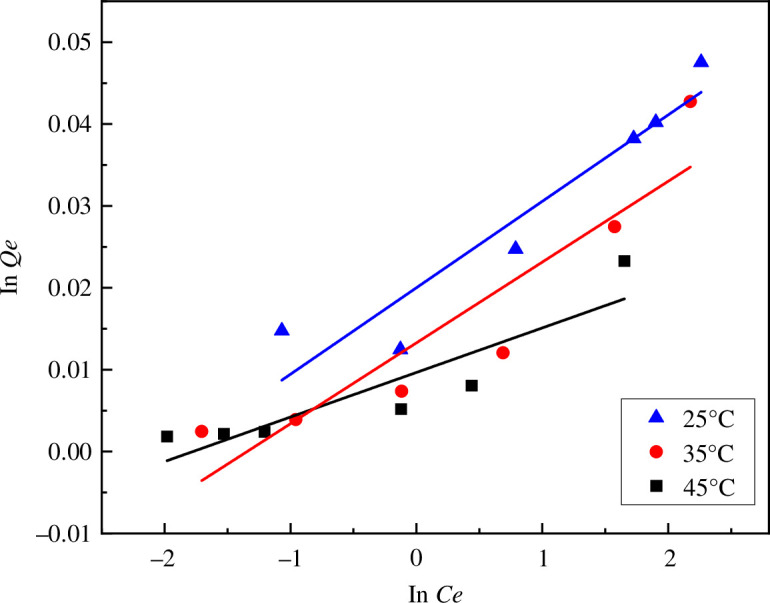
Freundlich isotherm of MACF@Fe_3_O_4_ adsorbing MB.

The adsorption kinetics of MB on MACF@Fe_3_O_4_ was investigated at 25°C, 35°C and 45°C. The common Langmuir model and Freundlich model were used for analysis. The results are shown in table 3, figures 21 and 22.

**Table 3.** Adsorption isotherm fitting parameters of MACF@Fe_3_O_4_ adsorbing MB.

temperature/ (℃)	Langmuir model			Freundlich model		
	*K*_L_ / (l·mg^−1^)	*Q*_m_ / (mg·g^−1^)	*R* ^2^	*K*_F_ / (mg^1−1/*n*^·l^1/*n*^·g^−1^)	1/*n*	*R* ^2^
25	6.1529	103.5197	0.9985	1.0202	0.0106	0.9035
35	4.5930	126.5823	0.9995	1.0134	0.0099	0.8511
45	3.2045	236.4066	0.9997	1.0097	0.0054	0.8179

From table 3, figures 21 and 22, it can be seen that the correlation coefficient R^2^ of the Langmuir model was larger than that of the Freundlich model, indicating that the adsorption of MB by MACF@Fe_3_O_4_ conformed to the Langmuir adsorption equation, indicating that the adsorption process was dominated by monolayer adsorption. The maximum theoretical saturated adsorption capacity was 236.41 mg g^−1^, and *Q*_m_ increases with the increase of temperature, indicating that the adsorption process was an endothermic process. The *R*_L_ values obtained by the Langmuir model at different temperatures were all less than 1.0, indicating that MACF@Fe_3_O_4_ had a good adsorption effect on MB, and the affinity between the two was large.

### 3.6. Adsorption thermodynamic analysis

In order to reveal the thermodynamic characteristics of the adsorption of neutral red by ferromagnetic coconut palm ACF, the Van't Hoff equation and thermodynamic relationship were used to plot $\frac{\text{1}}{\text{T}}$ with lnK_C_ and perform linear fitting. The corresponding Δ*S*°, Δ*H*° and Δ*G*° were calculated by the slope and intercept of the straight line, which are listed in table 4.

**Table 4.** Thermodynamic parameters of adsorption of MB by MACF@Fe_3_O_4_.

T /	Δ*G*° /	Δ*H*° /	Δ*S*° /
(K)	(KJ·mol^−1^)	(KJ·mol^−1^)	(J·mol^−1^·K^−1^)
298.15	−7.5516	27.90626	118.2237
308.15	−8.0770		
318.15	−9.9451		

According to table 4, The Δ*G*° < 0 at different temperatures (25–45°C) indicates that the adsorption process of MB by MACF@Fe_3_O_4_ was spontaneous. The value of Δ*G*° increased with the increase of temperature, indicating that the adsorption process at higher temperatures is credible. Δ*H*° > 0 indicating that the adsorption process was an endothermic process, and the temperature rise was conducive to the adsorption process. Δ*S*° > 0 indicates that the adsorption process of MACF@Fe_3_O_4_ on MB increased the confusion of the solid-liquid interface. This may be because when MB was adsorbed on MACF@Fe_3_O_4_, water molecules were desorbed from it, and the entropy caused by water molecule desorption increased more than the entropy reduction caused by MB adsorption, so the overall entropy was increased.

## 4. Discussion

This study is divided into three sections. Firstly, the ideal preparation and adsorption conditions of MACF@Fe_3_O_4_ were determined. Secondly, MACF@Fe_3_O_4_ was fully characterized, and the results confirmed the successful loading of nano-Fe_3_O_4_. Thirdly, the adsorption performance of MACF@Fe_3_O_4_ was studied, including adsorption kinetics, adsorption isotherm and adsorption thermodynamics. The high-value utilization of coconut palm fibre waste is realized, and nano-Fe_3_O_4_ endows it with excellent performance of convenient recycling. Therefore, MACF@Fe_3_O_4_ is expected to be industrially applied to urban sewage treatment, especially printing and dyeing wastewater treatment, and solve the problem of difficult recovery of powdered AC from a new perspective.

## Data Availability

The authors confirm that the data supporting the findings of this study are available within the article and its supplementary materials [35].
